# Comparative evaluation of ACRS and PRP on inflammation and lesion activity in a rat model of peritoneal endometriosis

**DOI:** 10.1186/s40001-026-03969-x

**Published:** 2026-02-05

**Authors:** Erol Karakaş, Mustafa Ermiş, Hanifi Erol, Gökhan Akçakavak, Nevzat Emre Aslan, Özhan Karataş

**Affiliations:** 1 Department of Obstetrics and Gynecology, Gynecology and Obstetrıcs Clinic, Kayseri, Turkey; 2 Experimental Research Application and Research Center, Department of Biochemistry, University of Erciyes, Kayseri, Turkey; 3 Faculty of Veterinary Medicine, Department of Surgery, University of Erciyes, Kayseri, Turkey; 4 Faculty of Veterinary Medicine, Department of Pathology, University of Aksaray, Aksaray, Turkey; 5 Faculty of Veterinary Medicine, Department of Surgery, University of Bozok, Yozgat, Turkey; 6 Faculty of Veterinary Medicine, Department of Pathology, University of Sivas Cumhuriyet, Sivas, Turkey

**Keywords:** Endometriosis, Autologous Cytokine-Rich Serum (ACRS), Platelet-Rich Plasma (PRP), Inflammation, Angiogenesis, TNF-α, IL-6, VEGFA, α-SMA, Rat

## Abstract

**Purpose:**

This study aimed to comparatively evaluate the therapeutic effects of Autologous Cytokine-Rich Serum (ACRS) and Platelet-Rich Plasma (PRP) in a rat model of endometriosis, focusing on inflammation, angiogenesis, and myofibroblast activity.

**Methods:**

A total of 36 adult female Wistar Albino rats were randomly assigned to six groups: healthy control, ACRS-only, PRP-only, endometriosis (EM), EM + ACRS, and EM + PRP. Endometriosis, modeled as lesion formation on the peritoneal wall, was surgically induced in the relevant groups. ACRS and PRP were prepared from autologous blood and administered intraovarianly and intraperitoneally. Lesions were excised for histopathological and immunohistochemical analysis of TNF-α, IL-6 (inflammation), VEGFA (angiogenesis), and α-SMA (fibrosis).

**Results:**

Histopathological scores decreased in both EM + ACRS and EM + PRP groups compared to the EM group. ACRS showed stronger anti-inflammatory effects, with lower TNF-α and IL-6 expression. However, ACRS-treated tissues also exhibited elevated VEGFA and α-SMA expression, suggesting increased angiogenesis and stromal activity.

**Conclusions:**

Both ACRS and PRP showed therapeutic effects. ACRS more effectively suppressed inflammation but may promote lesion stabilization through enhanced angiogenesis and fibrotic remodeling. These findings highlight the complex biological activity of ACRS, which requires further investigation before clinical translation.

## Introduction

Endometriosis is an estrogen-dependent, chronic inflammatory disease characterized by the presence of endometrial glands and stroma in ectopic locations outside the uterus, most commonly in the ovaries, peritoneum, and the Douglas pouch. Affecting approximately 10% of women of reproductive age, endometriosis leads to symptoms such as chronic pelvic pain, dysmenorrhea, dyspareunia, dyschezia, and infertility, all of which significantly impair quality of life. Additionally, the disease often shows progressive behavior, and a significant proportion of cases experience recurrence over time [1, 2].

Although the exact etiopathogenesis of endometriosis remains unclear, the most widely accepted hypothesis is Sampson’s theory of retrograde menstruation. According to this theory, endometrial cells shed during menstruation reflux into the peritoneal cavity via the fallopian tubes, where they implant ectopically. However, since retrograde menstruation occurs in nearly all women, the fact that only certain individuals develop endometriosis suggests that additional factors—such as immune intolerance, cellular adhesion and invasion capacity, an inflammatory microenvironment, and genetic/epigenetic predispositions—may play key roles [3, 4].

At the molecular level, endometriotic lesions are characterized by elevated expression of pro-inflammatory cytokines (e.g., TNF-α, IL-1β, IL-6), macrophage infiltration, increased angiogenesis (VEGF), and fibrogenesis [5–7]. Under the influence of cytokines, growth factors, and reactive oxygen species, the microenvironment of ectopic endometrial tissue gains proliferative and invasive characteristics, which contribute to disease persistence and progression [7, 8].

Current treatment strategies for endometriosis primarily include surgical excision and hormonal therapies. However, these approaches have notable limitations. Hormonal treatments often produce menopausal side effects (such as osteoporosis and vasomotor symptoms), making long-term use poorly tolerated, while surgical interventions are invasive and frequently associated with high recurrence rates. Therefore, there is a growing need for more targeted, innovative therapeutic alternatives with minimal side effects that can address the underlying pathogenesis of the disease.

In this context, regenerative medicine and biologic therapies have gained increasing attention. Platelet-Rich Plasma (PRP), an autologous product derived from whole blood and enriched with high concentrations of platelets, contains a variety of growth factors (PDGF, VEGF, EGF, TGF-β, IGF) and cytokines involved in wound healing, tissue regeneration, and modulation of inflammation. When applied locally, PRP promotes fibroblast proliferation, stimulates angiogenesis and extracellular matrix synthesis, and modulates immune responses. Currently, it is used therapeutically in various medical fields, including orthopedics, dermatology, urology, and gynecology [3, 4, 9].

Another promising biologic therapy developed in recent years is Autologous Cytokine-Rich Serum (ACRS), which is produced by incubating autologous blood under specific conditions for several hours, resulting in a serum enriched particularly with anti-inflammatory cytokines (IL-1ra, IL-4, and IL-10). ACRS is notably rich in IL-1 receptor antagonist (IL-1ra), which plays a critical role in suppressing the inflammatory response [5, 6]. Its clinical efficacy has been demonstrated in chronic inflammatory conditions such as osteoarthritis, musculoskeletal disorders, and neuropathic pain [5, 6].

The anti-inflammatory, antifibrotic, and regenerative properties of both PRP and ACRS align with the pathophysiological mechanisms of endometriosis [3, 4, 7, 8]. However, their potential effects in endometriosis, a complex and inflammation-driven disease, remain inadequately explored. Thus, investigating and comparing the therapeutic efficacy of PRP and ACRS in endometriosis from a molecular perspective is of considerable importance.

The aim of this study was to comparatively evaluate the effects of intraovarian administration of PRP and ACRS on inflammation (TNF-α, IL-6), angiogenesis (VEGFA), and fibrosis (α-SMA) in an experimentally induced model of endometriosis. In this context, the study analyzed the potential of these two therapeutic agents to modulate the molecular pathogenesis of endometriosis and explored their viability as novel preclinical treatment options [10].

It is important to note that the animal model used in this study mimics peritoneal lesion formation and does not represent the full systemic and hormonal aspects of human endometriosis.

## Materıals and methods

This study was conducted at the Erciyes University Experimental Research and Application Center (DEKAM) with the approval of the Erciyes University Local Animal Ethics Committee (HADYEK, Decision No: 24/023). All experimental procedures were carried out in accordance with international guidelines for the care and use of laboratory animals.

A study protocol was prepared prior to experimentation, but it was not registered in an external protocol registry.

### ARRIVE guidelines compliance statement

This study was conducted and reported in accordance with the ARRIVE (Animal Research: Reporting of In Vivo Experiments) guidelines (https://arriveguidelines.org). All procedures involving animals were reviewed and approved by the Erciyes University Animal Experiments Local Ethics Committee (HADYEK) on February 7, 2024 (Decision No: 24/023). Every effort was made to minimize animal suffering and the number of animals used.

Female Wistar Albino rats weighing between 200 and 300 g and aged 16–24 weeks were used in the study. The animals were obtained from Erciyes University DEKAM. They were housed in disinfected cages in groups of 4–5 per cage, with free access to standard rat chow and water (ad libitum). The animals were maintained under controlled environmental conditions with a 12-h light/dark cycle. All experimental procedures were performed at the same time of day to avoid circadian variations.

### Experimental groups

A total of 36 rats were randomly divided into six groups (*n* = 6 per group) as follows:

- Group I (Control group): No intervention was performed.
- Group II (PRP control group): Treated with platelet-rich plasma (PRP) only.
- Group III (ACRS control group): Treated with autologous cytokine-rich serum (ACRS) only.
- Group IV (Endometriosis–distilled water group): Endometriosis was induced, and distilled water was administered.
- Group V (Endometriosis–PRP group): Endometriosis was induced, and PRP treatment was administered.
- Group VI (Endometriosis–ACRS group): Endometriosis was induced, and ACRS treatment was administered.

Additionally, six rats were used as blood donors for the preparation of PRP and ACRS. Rats were randomly allocated to the groups using a computer-generated randomization sequence to reduce allocation bias. No a priori sample size calculation was performed; the group sizes were based on previous similar studies ensuring statistical relevance and ethical animal use. All animals survived until the end of the experiment and were included in the final analysis. No animals or data points were excluded.

### Endometriosis model

An experimental endometriosis model was established using the technique described by Vernon and Wilson [11], which has been shown in subsequent studies to closely mimic human endometriosis [12]. The model induced represents peritoneal endometriosis, as uterine tissue was autotransplanted onto the peritoneal wall to simulate ectopic implantation and lesion formation. General anesthesia was induced with intraperitoneal administration of ketamine (60 mg/kg) and xylazine (10 mg/kg) [12, 13]. Following anesthesia, the abdominal area was shaved and disinfected with povidone-iodine. A midline laparotomy was performed, and the right uterine horn was sutured to the peritoneal wall using 4/0 PDS to allow for autotransplantation [11]. After the procedure, the fascia and peritoneum were closed with 3/0 PDS sutures, and the skin was closed with 2/0 PGA. To allow for the formation of endometriotic lesions, animals were monitored for 4 weeks postoperatively [11, 12]. To confirm successful model induction, lesions were assessed macroscopically during necropsy and verified histologically by identifying ectopic endometrial glands, stroma, and hemosiderin-laden macrophages in the peritoneal wall—hallmark features of peritoneal endometriosis. Animals were monitored daily for food intake, weight loss, signs of pain or infection, wound healing, and overall behavior to ensure animal welfare and lesion development during the 4-week period.

### Preparation protocols for PRP

For PRP preparation, blood was collected from donor rats via intracardiac puncture under general anesthesia [12, 13] and transferred into tubes containing acid-citrate-dextrose (ACD) solution [1]. The samples were centrifuged at 1000 rpm for 10 min. After centrifugation, three layers formed: red blood cells at the bottom, a “buffy coat” layer in the middle containing platelets, leukocytes, and a small amount of erythrocytes, and platelet-poor plasma at the top. The plasma fraction was carefully aspirated up to the interface layer using a pipette and transferred into a separate sterile tube [1, 8]. Approximately, 6 ml of PRP was obtained from 10 ml of whole blood [1, 8].

### Preparation of ACRS

For the preparation of Autologous Cytokine-Rich Serum (ACRS), intracardiac blood was collected from donor rats under general anesthesia and transferred into specialized kit tubes. The blood samples were incubated in a dedicated incubator for 3 h, followed by centrifugation at 4000 rpm for 5 min [5, 6]. After centrifugation, the serum formed at the top was carefully aspirated using a pipette and transferred into another tube. Approximately, 6 mL of ACRS was obtained from 10 mL of blood [5, 6].

### Treatment protocols

In Groups II and III, the rats underwent laparotomy under general anesthesia [12, 13], and a single dose of 0.5 mL of PRP or ACRS was administered directly into the right ovary via intraovarian injection [1, 5]. This was followed by two additional intraperitoneal injections of 0.5 mL PRP or ACRS at one-week intervals [1]. The right ovary was selected for the initial intraovarian injection due to its accessibility and to allow direct exposure of ovarian tissue to therapeutic agents. Subsequent intraperitoneal injections were performed to ensure uniform distribution throughout the abdominal cavity and interaction with peritoneal lesions.

In Groups IV, V, and VI, after endometriosis model induction and a 4-week period for lesion development [11, 12], a laparotomy was performed under general anesthesia [12, 13]. A single dose of 0.5 mL of distilled water (Group IV), PRP (Group V), or ACRS (Group VI) was administered intraovarianly to the right ovary [1, 5]. Subsequently, two additional doses of 0.5 mL distilled water, PRP, or ACRS were administered intraperitoneally at one-week intervals [1, 5].

The right ovary was selected due to its consistent anatomical accessibility, and intraperitoneal injections were used to ensure broader agent distribution across the abdominal cavity.

At the end of the study (Day 31 for the control groups and Day 45 for the endometriosis groups), all rats were sacrificed under anesthesia with xylazine (10 mg/kg) and ketamine (60 mg/kg) via cervical dislocation [12]. The abdominal cavities were opened, and endometriotic lesions and relevant tissue samples were collected for histopathological and immunohistochemical analysis [11, 12, 14].

### Histopathological and immunohistochemical analysis

Uterine tissue samples collected post-necropsy were fixed in 10% neutral buffered formalin [12]. Following fixation, tissues underwent routine histological processing and were embedded in paraffin blocks. Sections of 4–5 μm thickness were cut from the paraffin blocks, stained with hematoxylin and eosin (H&E), and examined under a light microscope (Olympus BX51, Tokyo, Japan) [12, 13, 25].

For the histopathological evaluation, the endometriosis grading system previously described was employed [11, 12, 14, 26]. According to this system, tissue samples were semi-quantitatively scored by a blinded pathologist as follows:

- 0: No epithelial lining
- 1: Poorly preserved epithelium (epithelial cells present only occasionally)
- 2: Moderately preserved epithelium with leukocyte infiltration
- 3: Well-preserved epithelial layers

For immunohistochemical analysis, sections from paraffin blocks were subjected to deparaffinization and rehydration. Immunohistochemical staining was performed using a commercial kit (UltraVision Large Volume Detection System Anti-Polyvalent, HRP, TP-060-HL) according to the manufacturer’s instructions [12]. The following primary antibodies were used:

- α-SMA (alpha-smooth muscle actin, Affinity Biosciences, AF1032, dilution 1:200) [8]
- TNF-α (Tumor necrosis factor-alpha, Santa Cruz Biotechnology, sc-133192, dilution 1:400) [8]
- IL-6 (Interleukin-6, Affinity Biosciences, DF6087, dilution 1:400) [8]
- VEGFA (Vascular Endothelial Growth Factor A, Affinity Biosciences, AF5131, dilution 1:200) [8]

DAB (3,3′-diaminobenzidine) was used as the chromogen, and counterstaining was performed with Mayer’s hematoxylin [12]. Immunohistochemical scoring was conducted semi-quantitatively by a blinded pathologist based on staining intensity and extent: 0 (none), 1 (mild), 2 (moderate), and 3 (strong) [27].

Histopathological and immunohistochemical evaluations were performed by a pathologist blinded to group allocation. All treatments were administered at the same time of day to avoid circadian bias. Cage conditions were standardized across groups.

### Statistical analysis

Statistical analysis of data was performed using the nonparametric Kruskal–Wallis test. Pairwise comparisons between groups were conducted using the Mann–Whitney *U* test. A *p*-value < 0.05 was considered statistically significant. All analyses were performed using appropriate statistical software.

## Results

**Fig. 1:**
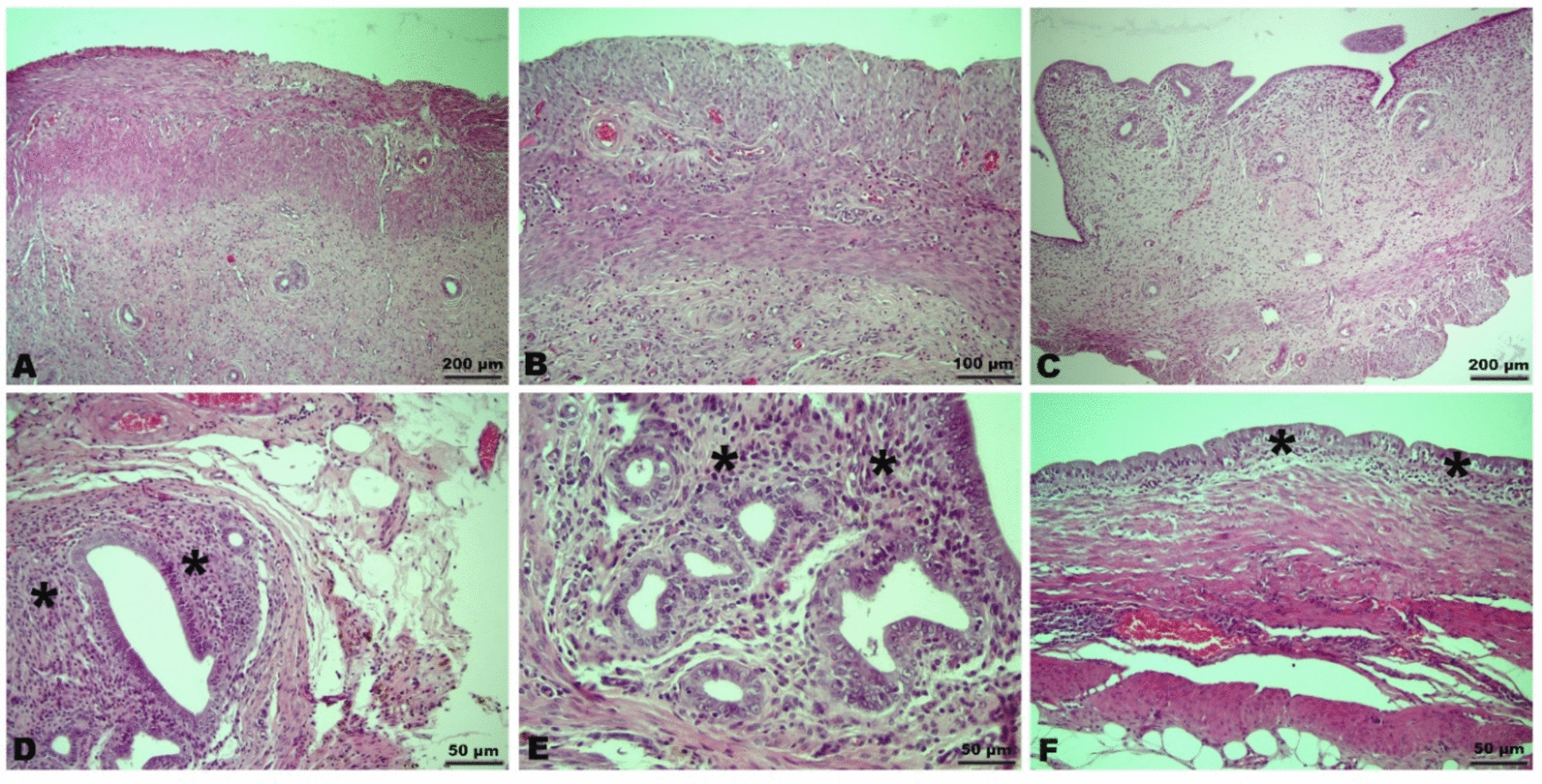
Microscopic evaluation of samples from different groups stained with Hematoxylin–Eosin (H&E). **A**–**B**–**C** Representative images of control groups (Group I, Group II, Group III) showing a score of 3 in the endometriosis grading system. **D** Image of Group IV showing a score of 0. **E** Image of Group V showing a score of 2. **F** Image of Group V showing a score of 2. (Asterisk; inflammatory cell infiltration)

Histopathological examination revealed that uterine tissues in the control groups (Groups I, II, and III) exhibited normal histological architecture (Fig. 1A, B, C). In the endometriosis-induced groups (Groups IV, V, and VI), glandular epithelial hyperplasia, secretions within endometrial gland lumens, inflammatory cell infiltration in the stroma, and numerous macrophages with hemosiderin-laden cytoplasm were observed (Fig. 1D, F). In the endometriosis-ACRS group (Group VI), the frequency and severity of lesions were found to be reduced compared to the endometriosis-distilled water group (Group IV).

Endometriosis grading scores were lowest in Group IV compared to the control groups (Groups I, II, and III), and this difference was statistically significant. In Group VI, which received ACRS treatment, endometriosis scores were significantly increased compared to Group IV (Table 1).

**Table 1:** Endometriosis grading and immunohistochemical scores among groups (Mean ± SE)

	Group I	Group II	Group III	Group IV	Group V	Group VI
Endometriosis grading	3.00 ± 0.00^a^	2.83 ± 0.17^a^	2.83 ± 0.17^a^	1.33 ± 0.21^c^	1.67 ± 0.21^c^	2.33 ± 0.21^ab^
α-SMA	1.83 ± 0.30^ab^	2.00 ± 0.36^a^	2.00 ± 0.36^a^	1.33 ± 0.21^b^	1.50 ± 0.22^b^	2.33 ± 0.21^a^
VEGFA	1.67 ± 0.33^ab^	1.83 ± 0.40^a^	1.83 ± 0.30^a^	1.17 ± 0.17^b^	1.33 ± 0.21^b^	2.17 ± 0.30^a^
IL-6	0.17 ± 0.17^c^	0.33 ± 0.21^c^	0.50 ± 0.22^c^	2.50 ± 0.22^a^	1.83 ± 0.17^b^	1.33 ± 0.21^b^
TNF- α	0.33 ± 0.21^c^	0.33 ± 0.21^c^	0.50 ± 0.22^c^	2.33 ± 0.21^a^	1.67 ± 0.21^b^	1.17 ± 0.17^b^

^a^^−^^d^Letters in the same row indicate statistically significant differences between groups (*p* < 0.001)

**Fig. 2:**
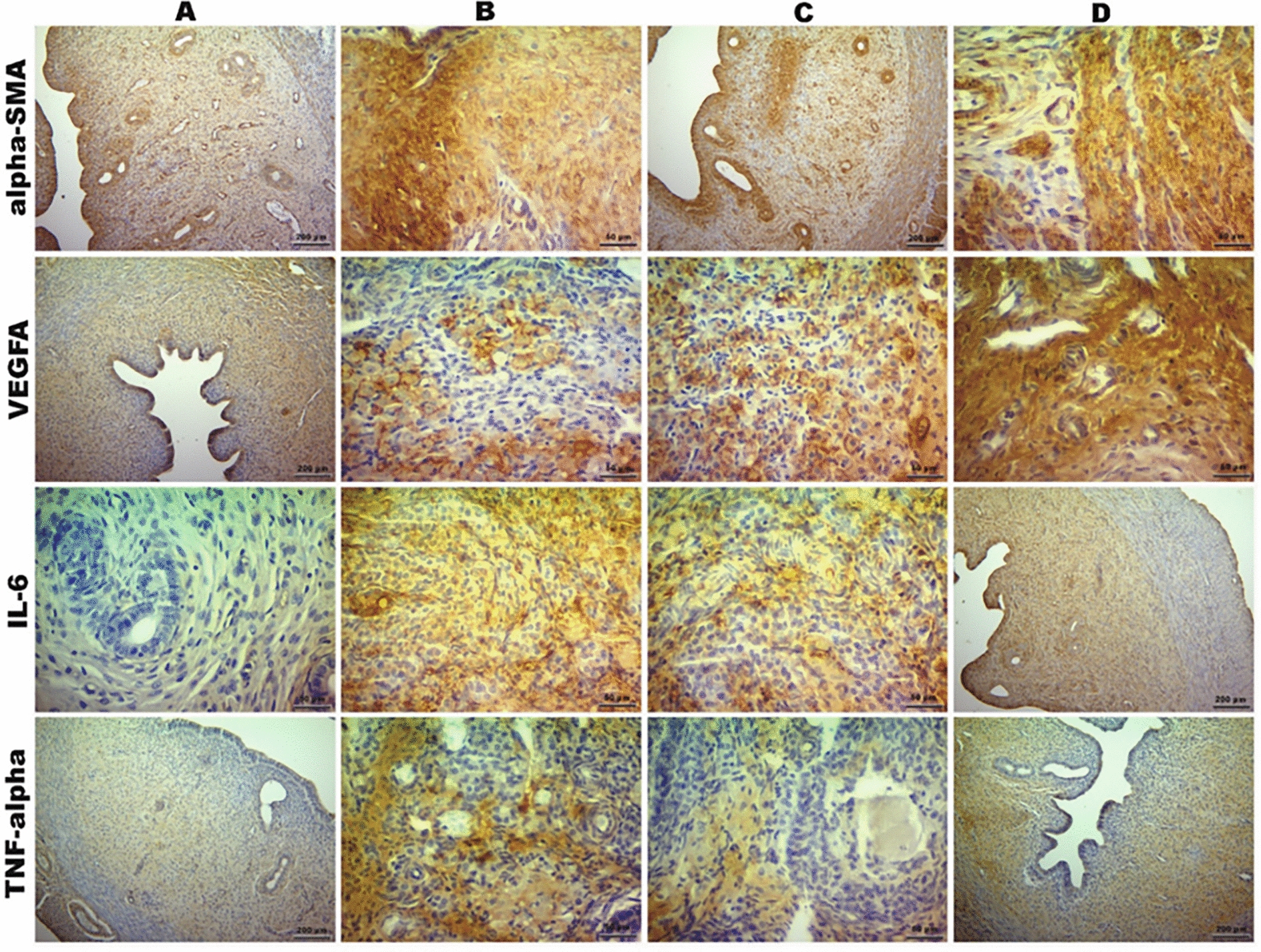
Immunohistochemical expression of α-SMA, VEGFA, IL-6, and TNF-α across groups (DAB staining). (**A**; Group I, **B**; Group IV, **C**; Group V, **D**; Group VI)

Immunohistochemical staining (Fig. 2) showed that VEGFA and α-SMA expression scores were lowest in Group IV and highest in Group VI. ACRS treatment in Group VI significantly increased VEGFA and α-SMA immunoreactivity compared to Group IV, indicating that ACRS therapy enhances angiogenesis and myofibroblast activity.

For TNF-α and IL-6 immunostaining, the lowest scores were observed in the control groups (Groups I, II, and III), whereas the highest scores were detected in the endometriosis-distilled water group (Group IV). Both PRP (Group V) and ACRS (Group VI) treatments significantly reduced TNF-α and IL-6 expressions compared to Group IV. This reduction was more pronounced in the ACRS-treated group. These findings suggest that ACRS treatment may be more effective than PRP in suppressing the inflammatory response.

## Discussion

Endometriosis is a complex, estrogen-dependent inflammatory disease that causes significant symptoms such as chronic pelvic pain, dysmenorrhea, and infertility in women, negatively affecting quality of life. The multifactorial etiology of the disease and its tendency to recur often lead to the inadequacy of current treatment approaches. Although surgical excision and hormonal therapies aim to suppress the disease, their long-term success rates are relatively low, and recurrent symptoms are frequently observed in many cases [15]. In this context, regenerative medicine-based biological treatment approaches such as platelet-rich plasma (PRP) and autologous cytokine-rich serum (ACRS) have attracted attention for their potential in modulating inflammation and supporting tissue regeneration [16]. In this study, the effects of ACRS and PRP treatments on an experimental endometriosis model were evaluated comparatively. Both treatments demonstrated therapeutic effects on the lesions; however, these effects appear to occur through different molecular mechanisms.

### Effects on the inflammatory response

It is well established that immune dysregulation and chronic inflammation play a central role in the pathogenesis of endometriosis [17]. Particularly, elevated levels of pro-inflammatory cytokines such as TNF-α and IL-6 in peritoneal fluid and endometriotic lesions emphasize the importance of inflammation in disease progression [18]. In our study, significantly increased levels of these cytokines were observed in the untreated group with induced endometriosis, confirming the pathophysiological validity of the model. Both ACRS and PRP treatments significantly reduced TNF-α and IL-6 expression levels, indicating that both biological agents have anti-inflammatory potential. However, the decrease was more pronounced in the ACRS-treated group, suggesting that ACRS may have a stronger anti-inflammatory effect. This could be attributed to the presence of potent anti-inflammatory cytokines in ACRS, such as IL-1 receptor antagonist (IL-1Ra), IL-4, and IL-10 [19]. These findings suggest that ACRS may be a promising treatment option targeting the inflammatory component of endometriosis, potentially providing symptomatic relief.

### Effects on angiogenesis and myofibroblast activity

The persistence of endometriotic lesions is not only associated with inflammation but also with processes such as angiogenesis and fibrosis [20]. VEGFA has been identified as a key factor responsible for the vascularization of endometriotic foci. α-SMA is an indicator of myofibroblast activity and fibrotic tissue remodeling, and it may play a role in the chronic persistence of lesions [21]. In our study, ACRS treatment significantly increased both VEGFA and α-SMA expression, indicating angiogenic and fibrotic activity. Although VEGFA and α-SMA levels increased in the ACRS-treated group, this pattern may represent regenerative or stabilizing effects rather than true lesion regression. Therefore, the observed enhancement in angiogenesis and stromal activity should be interpreted with caution, as such responses may potentially contribute to lesion persistence in the context of endometriosis. These findings suggest that ACRS has a complex biological role, capable of suppressing inflammation but potentially enhancing angiogenic and stromal activity [22]. This dual nature underscores the need for cautious interpretation and further studies to determine whether ACRS promotes long-term lesion regression or inadvertently supports their maintenance.

### Clinical implications and evaluation of treatment options

Histopathological scoring of endometriosis revealed significant differences among treatment groups. Particularly, the higher scores observed in the ACRS group may reflect preservation of lesion structure, but this could indicate incomplete regression rather than therapeutic resolution. This may point to ACRS’s tissue-supportive effects; however, it should also be considered that such protective effects might hinder lesion regression.

Estrous cycle staging via vaginal cytology was not performed, which is a limitation as hormonal phase may influence lesion development and treatment response. Lesion growth was not monitored via imaging during the study period, and lesion size or weight was not quantified, which limits the depth of lesion response assessment. From a clinical perspective, ACRS may contribute to symptom relief by suppressing inflammatory processes, while PRP, due to its more limited effect on angiogenesis and balanced cytokine profile, might present a safer and more conservative therapeutic option that is less likely to promote lesion persistence [23]. Based on these results, PRP could be considered a more balanced and safer biological agent for endometriosis treatment, whereas ACRS may be preferable in cases where inflammation suppression is a primary goal. Nonetheless, these suggestions require further support through advanced preclinical and clinical studies on human tissues before being integrated into clinical practice.

### Study limitations and future perspectives

Another limitation was the lack of baseline lesion grading prior to treatment initiation. Potential variability in initial lesion size or severity across groups may have contributed to differences in treatment outcomes, particularly the unexpected higher histological scores in Group VI. The limitations of this study include the small sample size, the focus on only specific biomarkers, and the lack of evaluation of the long-term effects of the treatments. Additionally, the experimental model used in this study only partially reflects human pathophysiology, limiting the generalizability of the findings [24]. Future research should compare different doses and administration regimens of ACRS and PRP and evaluate broader inflammatory, fibrotic, and angiogenic responses at the molecular level. Furthermore, investigating the effects of these treatments on fertility, endometrial receptivity, and oocyte quality is essential for potential clinical translation.

## Conclusion

This study demonstrates that both ACRS and PRP have anti-inflammatory effects in an experimental peritoneal endometriosis model. ACRS more effectively reduced TNF-α and IL-6 levels; however, it also increased markers of angiogenesis (VEGFA) and stromal remodeling (α-SMA), which could potentially stabilize lesions rather than resolve them. These findings suggest that while ACRS holds promise in modulating inflammation, its effects on tissue remodeling must be carefully evaluated. PRP, by contrast, may provide a more balanced therapeutic profile with less stimulation of angiogenesis or fibrosis. Further studies are necessary to clarify the long-term effects and optimal therapeutic context for each treatment modality.

## Key findings

- Both ACRS and PRP treatments reduced the inflammatory response in an experimental endometriosis model.
- ACRS more effectively suppressed TNF-α and IL-6 expression compared to PRP, while also increasing VEGFA and α-SMA expression levels.
- These findings suggest that ACRS plays an active role not only in inflammation control but also in angiogenesis and stromal remodeling processes.

## Clinical applications

- The data suggest that ACRS and PRP could be considered complementary or alternative treatment options, especially in cases resistant to conventional therapies or in recurrent endometriosis.
- ACRS, with its potential to modulate immune response and support tissue regeneration, may hold a significant place in future personalized treatment strategies for endometriosis.

## Future research

- The long-term effects of ACRS and PRP, as well as optimal dosages and application frequencies, need to be established.
- Evaluating the efficacy of these biological therapies in combination with hormonal treatments or surgical interventions may be crucial in developing next-generation endometriosis treatment protocols.
- It is recommended that future studies explore their molecular effects, particularly on the TGF-β pathway, macrophage polarization, ECM remodeling, and epithelial-mesenchymal transition (EMT).

## Data Availability

All the data are included in the manuscript.
